# Hippocampal delivery of neurotrophic factor-α1/carboxypeptidase E gene prevents neurodegeneration, amyloidosis, memory loss in Alzheimer’s Disease male mice

**DOI:** 10.1038/s41380-023-02135-7

**Published:** 2023-06-28

**Authors:** Lan Xiao, Xuyu Yang, Vinay Kumar Sharma, Daniel Abebe, Y. Peng Loh

**Affiliations:** Section on Cellular Neurobiology, Eunice Kennedy Shriver National Institute of Child Health and Human Development, National Institutes of Health, Bethesda, Md 20892 USA

**Keywords:** Neuroscience, Physiology

## Abstract

Alzheimer’s Disease (AD) is a prevalent neurodegenerative disease characterized by tau hyperphosphorylation, Aβ1-42 aggregation and cognitive dysfunction. Therapeutic agents directed at mitigating tau aggregation and clearing Aβ1-42, and delivery of growth factor genes (BDNF, FGF2), have ameliorated cognitive deficits, but these approaches did not prevent or stop AD progression. Here we report that viral-(AAV) delivery of Neurotrophic Factor-α1/Carboxypeptidase E (NF-α1/CPE) gene in hippocampus at an early age prevented later development of cognitive deficits as assessed by Morris water maze and novel object recognition assays, neurodegeneration, and tau hyperphosphorylation in male 3xTg-AD mice. Additionally, amyloid precursor protein (APP) expression was reduced to near non-AD levels, and insoluble Aβ1-42 was reduced significantly. Pro-survival proteins: mitochondrial Bcl2 and Serpina3g were increased; and mitophagy inhibitor Plin4 and pro-inflammatory protein Card14 were decreased in AAV-NF-α1/CPE treated versus untreated AD mice. Thus NF-α1/CPE gene therapy targets many regulatory components to prevent cognitive deficits in 3xTg-AD mice and has implications as a new therapy to prevent AD progression by promoting cell survival, inhibiting APP overexpression and tau hyperphosphorylation.

## Introduction

Alzheimer’s Disease (AD) is the most prevalent form of dementia, currently affecting >50 million people globally, and is expected to triple by 2050 [1]. This disease occurs mainly in patients over 65 years of age and is marked by memory loss which is subtle in the beginning and worsens over time. It is a complex disorder determined by environmental factors throughout life, and in <1% of cases, genetically inherited, where cognitive impairment occurs in early adulthood [2]. Familial AD is primarily attributable to mutations of one of three genes: *Amyloid precursor protein,(APP), presenilins 1* and *2* [2–4]. Other gene modifications such as a variant of *apolipoprotein*, *(APOEε4), TOMM40* and *EPC2* have been associated with high risk of developing AD [5, 6].

AD is a neurodegenerative disease characterized by pathology that includes hyperphosphorylation of tau, leading to neurofibrillary tangles, and deposition of aggregated amyloid β1-42 to form senile plaques [7, 8]. While it is believed that tangles and plaques found in AD patients are responsible for neurodegeneration, their causal effects remain unclear. Various therapeutic agents designed to ameliorate AD progression have been directed at amyloid clearance, targeting tau aggregation and decreasing neuroinflammation [9–11]. Although some of these approaches have shown success in improving spatial learning and memory in transgenic AD mouse models, and mitigating AD symptoms in patients, none have prevented, stopped or delayed disease progression [12–14].

Neurotrophic factors such as nerve growth factor (NGF), brain derived neurotrophic factor (BDNF) and Fibroblast growth factor 2 (FGF2) have been shown to promote neuronal survival, maturation [15] and neurogenesis [16]. Hence, delivery of neurotrophic factor genes or protein to the brain of AD patients offers a different approach to AD therapy [17]. Adeno-associated viral-(AAV) NGF gene delivery in a Phase I clinical trial on AD patients appeared to have shown improvement in cognitive decline [18]. Treatment of amyloid-transgenic mice by injection of lentivirus-BDNF into hippocampal entorhinal cortex after disease onset prevented cell death and ameliorated cognitive deficits, independent of direct modulation of amyloid [19]. This finding indicated the efficacy of BDNF gene therapy, leading to a clinical trial delivering AAV-BDNF into AD patients which is ongoing [20]. In another study, AAV-FGF2 gene delivery into the hippocampus of APP+presenilin-1bigenic mice at the pre- and post-symptomatic stage reversed learning deficits, partially improved clearance of amyloid β peptide only in the pre-symptomatic group and enhanced neurogenesis in the dentate gyrus [21].

Recently, Neurotrophic Factor-α1 (NF-α1), originally known as Carboxypeptidase E (CPE) was identified and shown to have a number of trophic effects [22, 23], independent of its enzymatic activity; most notably in protecting neurons from stress-induced cell death in the hippocampal CA3 region in mice and was more critical than BDNF in this respect [24]. Studies using a mouse model expressing a non-enzymatic form of NF-α1/CPE demonstrated that it was as effective in neuroprotection as wild-type NF-α1/CPE [24]. In CPE-knock-out mice, severe stress resulted in complete degeneration of CA3 hippocampus and cognitive dysfunction despite having normal levels of BDNF [24, 25]. After global ischemia, increased and sustained NF-α1/CPE expression in CA3 hippocampal neurons facilitated their survival in rats [26].

Secreted NF-α1/CPE mediates its neuroprotective effect by interacting with human serotonin receptor, HTR1E, to activate the Erk-Creb signaling pathway in an arrestin-dependent manner, leading to increased expression of Bcl2, a mitochondrial pro-survival protein [27]. A mutation consisting of three adenosine inserts in the CPE gene identified in an AD patient has been shown to cause memory deficit and depression-like behavior in transgenic mice [28]. In the normal human hippocampus, immunoreactive NF-α1/CPE was found in neuronal cell bodies and plasma membrane where it co-localized with HTR1E [27]. In human cortex, NF-α1/CPE immunoreactivity was detected in neuronal cell bodies and processes, and in astrocytes [29]. In contrast, in the brains of AD patients, NF-α1/CPE immunostaining was found primarily in senile plaques [29]. The strong neuroprotective function of NF-α1/CPE, prompted us to investigate whether AAV- NF-α1/CPE gene delivery to the hippocampus could prevent AD progression in mice. Here we used the 3xTg-AD Alzheimer’s disease mouse model for our studies. These mice harbor 3 human mutant alleles, homozygous for *Psen1* mutation, PS1M146V, and two transgenes, APP with Swedish mutation, APPSwe, and tauP301L mutation [30]. They exhibit age-related cognitive impairment, extracellular amyloid plaques and tau pathology. Our study demonstrates that hippocampal AAV-NF-α1/CPE treatment rescues neurodegeneration, cognitive dysfunction, tau hyperphosphorylation and elevated amyloid precursor protein (APP)/Aβ42 expression in male 3xTg-AD mice.

## Results

### Long-Term NF-α1/CPE expression in hippocampus after gene delivery in 3xTg-AD mice

**Fig. 1:**
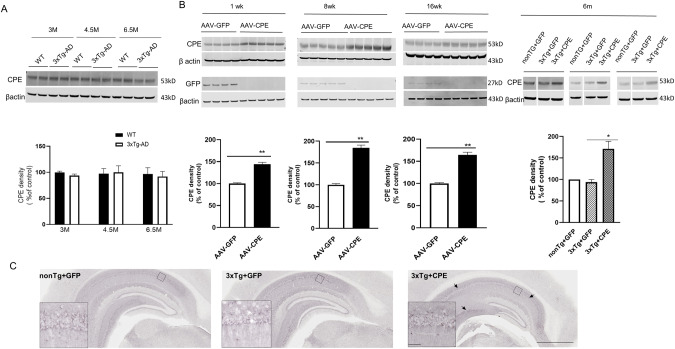
CPE expression in hippocampus of 3xTg-AD mice after AAV-NF-α1/CPE injection. **A** Hippocampal CPE expression in WT and 3xTg-AD mice at age of 3, 4.5 and 6.5 months. **B** Hippocampal CPE expression 1wk, 8wk, 16wk, and ~6 months after stereotaxic injection of AAV-NF-α1/CPE, or AAV-GFP into hippocampus of 2 months old 3xTg-AD mice. (Left panel) CPE expression was significantly increased in 3xTg+CPE in comparison with 3xTg+GFP group. *n* = 4–5 mice, *t*-test, **p < 0.01 compared with GFP control, values are mean ± SEM. (Right panel) Hippocampal CPE expression at ~8 months old after stereotaxic injection in nonTg+GFP, 3xTg+GFP and 3xTg+CPE at 2 months of age. One-way ANOVA analysis followed by Tukey’s *post-hoc* multiple comparison test, [*F*_(2,12)_ = 16.47, *p* = 0.0004]. *p = 0.0006 for 3xTg+CPE compared with 3xTg+GFP, *n* = 5 per each genotype. Values are mean ± SEM. **C** Representative immunohistochemistry images showing CPE expression after stereotaxic injection of AAV-NF-α1/CPE or AAV-GFP in the hippocampus of nonTg+GFP, 3xTg+GFP, and 3xTg+CPE mice at ~8 months of age. Scale bar = 1 mm. Arrows show areas of increased CPE immunoreactivity in 3xTg-CPE mice. Inset: scale bar = 50 μm.

First, we examined NF-α1/CPE protein levels in the hippocampus, an area of the brain with one of the highest expression, in 3xTg-AD and wild type (WT) mice. Figure 1A shows that at 3, 4.5 and 6.5 mths of age there was no significant difference in expression between these 2 genotypes. We then analyzed the long term expression of NF-α1/CPE in the hippocampus of 3xTg-AD mice after NF-α1/CPE gene delivery. AAV-NF-α1/CPE or AAV-GFP (as negative control) was injected bilaterally into the hippocampus (see Supplementary Fig. S1A for injection site) of 3xTg-AD mice at 2 months of age. A control comparing APP and phosphorylated tau levels in the hippocampus of 3xTg-AD mice 4 months after injection with AAV-GFP, and uninjected age-matched 3xTg-AD mice showed no difference, indicating that the injection procedure did not enhance AD pathology in these mice (Supplementary Fig. S1B, C). Analysis at 1, 8, 16 weeks and ~6 months after gene delivery, mice injected with AAV-NF-α1/CPE showed an ~1.5–1.8 fold higher levels of NF-α1/CPE protein in hippocampus versus AAV-GFP injected mice, at all ages studied, (Fig. 1B). Furthermore at ~8 months of age, (6 months after AAV-CPE injection), NF-α1/CPE expression in 3xTg-AD-CPE mice was ~1.7 fold higher than non-Tg-GFP mice and 3xTg-AD-GFP mice, indicating sustained long term expression. Morphologically, the cell pattern of over-expression of NF-α1/CPE in the hippocampi of ~8-month-old 3xTg-AD-CPE mice was very similar to both 3xTg-AD-GFP and non-Tg-GFP mice apart from the increased intensity of CPE immunostaining (Fig. 1C and insets). Since CPE is an obesity susceptibility gene [31], we also monitored the weight of these animals and found no difference in weight between the 2 genotypes with or without AAV-NF-α1/CPE treatment at 2 months or 7–8 months of age (Supplementary Fig. S2A, B).

### NF-α1/CPE gene delivery in hippocampus rescues memory deficits in 3xTg-AD mice

**Fig. 2:**
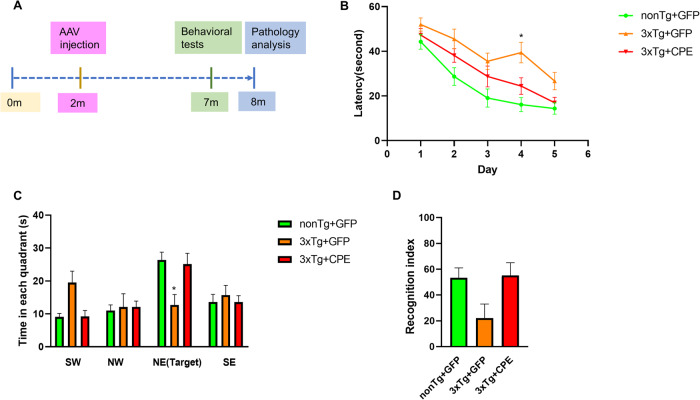
AAV-NF-α1/CPE gene delivery in hippocampus prevents memory loss in 3xTg-AD mice. **A** Experimental design for AAV injection followed by behavioral and pathological analyses of 3xTg-AD mice. 3xTg-AD mice received bilateral hippocampal injections of AAV-GFP or NF-α1/CPE at age 2 months and were evaluated by a series of behavioral tests at age of 7 months. Pathology of the hippocampus of the mice was examined at age ~8 months. **B** The effect of overexpression of NF-α1/CPE on spatial learning in Morris water maze. 3xTg+GFP mice displayed longer latency compared with nonTg+GFP on day 2, day 3, and day 4, *p* = 0.0048 for day 2, *p* = 0.0061 for day 3, *p* < 0.0001 for day 4, *p* = 0.0547 for day 5. 3xTg+CPE mice showed no significant difference in latency compared to 3xTg+GFP mice, except on day 4, *p* = 0.0103 and a trend on day 5. Two-way ANOVA analysis followed by Tukey’s post-hoc multiple comparison test, for factor day [*F*_(4,170)_ = 27.86, *p* < 0.0001]. For factor genotype [*F*_(2,170)_ = 21.11, *p* < 0.0001]. Day × Genotype: [*F*_(8,170)_ = 0.7515, *p* = 0.6459]. *n* = 12 for nonTg+GFP, *n* = 11 for 3xTg+GFP, *n* = 14 for 3xTg+CPE. Values are mean ± SEM. **C** Overexpression of NF-α1/CPE prevented memory deficit of 3xTg-AD mice in Morris water maze test. 3xTg+GFP mice spent less time in the target area NE, and more time in non-target areas. Both nonTg+GFP and 3xTg+CPE mice displayed similar pattern of time in non-target quadrants and target quadrant. In NE target quadrant, 3xTg+CPE mice spent more time, similar to nonTg+GFP mice, than 3xTg+GFP mice. *p = 0.0025 for 3xTg+CPE compared with 3xTg+GFP. Two-way ANOVA analysis followed by Tukey’s post-hoc multiple comparison test, for factor quadrant [*F*_(3,136)_ = 8.691, *p* < 0.0001]. For factor genotype [*F*_(2,136)_ = 5.942e−006, *p* > 0.9999]. Quadrant × Genotype: [*F*_(6,136)_ = 4.475, *p* = 0.0004]. *n* = 12 for nonTg+GFP, *n* = 11 for 3xTg+GFP, *n* = 14 for 3xTg+CPE. Values are mean ± SEM. **D** Effect of overexpression of NF-α1/CPE on cognitive function of 3xTg AD mice in novel object recognition test. ANOVA analysis followed by Tukey’s post-hoc multiple comparison test showed no significant differences across groups, but a trend showing memory improvement in 3xTg-AD mice. *n* = 10 for nonTg+GFP, *n* = 8 for 3xTg+GFP, *n* = 12 for 3xTg+CPE. Values are mean ± SEM.

The ages at which 3xTg-AD and non-Tg (control) mice were bilaterally injected into the hippocampus with AAV-NF-α1/CPE or AAV-GFP and when behavioral and neuropathological analysis were carried out are displayed in Fig. 2A. At 2 months of age, 3xTg-AD mice are pre-symptomatic, but by 6 months of age, they exhibit cognitive dysfunction and AD neuropathology [32]. Cognitive function in treated animals was evaluated at 7 months of age. Analysis of spatial learning and memory functions in the Morris water maze test showed mild learning deficits (Fig. 2B), but severe memory (Fig. 2C) loss in the 3xTg-AD mice. The memory deficit was prevented in 3xTg-AD mice overexpressing CPE which were comparable to non-Tg mice. Figure 2D shows the results of the Novel Object Recognition test in 3xTg-AD mice with and without AAV-NF-α1/CPE treatment and non-Tg mice. While ANOVA analysis did not show any significant differences across the groups, the data clearly support a trend that the object recognition ability was decreased in 3xTg+GFP in comparison with nonTg+GFP mice, suggesting object recognition deficit in 3xTg-AD mice. Moreover, the data also suggest a trend that AAV-NFα1/CPE treatment prevented memory deficit in novel object recognition in 3xTg mice. The object recognition index of NF-α1/CPE treated 3xTg-AD mice was comparable to non-Tg-GFP control mice (Fig. 2D). The open field test showed no difference in distance and speed traveled between the 3xTg-GFP, 3xTg-CPE and non-Tg-GFP mice (Supplementary Fig. S2C, D). The elevated plus maze test did not reveal any difference in anxiety-like behavior between the 3xTg-GFP, 3xTg -CPE and non-Tg-GFP mice (Supplementary Fig. S2E, F). Interestingly, in the forced swim test for depression-like behavior, there was a trend towards a decrease in immobility time in 3xTg-AD mice whether over-expressing CPE or not, relative to non-Tg mice (Supplementary Fig. S2G).

### AAV-NF-α1/CPE gene delivery prevents hippocampal neurodegeneration in 3xTg-AD mice

**Fig. 3:**
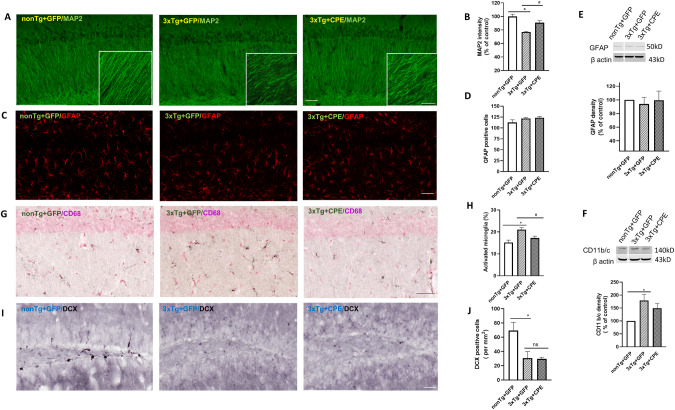
AAV-NF-α1/CPE gene delivery prevents hippocampal neurodegeneration in 3xTg-AD mice. **A** Representative sections of MAP2 immunofluorescence staining of hippocampal CA1 region of nonTg+GFP, 3xTg+GFP and 3xTg+CPE mice at age ~8 months. Magnification 10x, scale bar =50 μm., Inset: magnification 20x, scale bar =50 μm. n = 6 mice per genotype. **B** Quantification of MAP2 intensity in CA1 region of nonTg+GFP, 3xTg+GFP and 3xTg+CPE mice at age ~8 months. MAP2 intensity was decreased in 3xTg+GFP mice in comparison with nonTg+GFP, *p = 0.0002; 3xTg+CPE increased MAP2 intensity significantly in comparison with 3xTg+GFP, ^#^*p* = 0.0129. One-way ANOVA analysis followed by Tukey’s post-hoc multiple comparison test, [*F*_(2,15)_ = 15.45, *p* = 0.0002]. Values are mean ± SEM. 4 sections per mouse, *n* = 6 mice per genotype. nonTg+GFP made = 100% as control. **C** Representative sections of GFAP immunofluorescence staining of hippocampal CA1 of nonTg+GFP, 3xTg+GFP and 3xTg+CPE mice at age ~8 months. Magnification 10x, scale bar = 50 μm. *n* = 6 mice per genotype. **D** Quantification of GFAP positive cells in CA1 region of nonTg+GFP, 3xTg+GFP and 3xTg+CPE mice at age ~8 months. One-way ANOVA analysis followed by Tukey’s post-hoc multiple comparison test did not show any significant differences. Values are mean ± SEM. 4 sections per mouse, *n* = 6 mice per genotype. **E** Representative Western blot and quantification of GFAP expression in hippocampus of nonTg+GFP, 3xTg+GFP and 3xTg+CPE mice at age ~8 months. One-way ANOVA analysis followed by Tukey’s post-hoc multiple comparison test did not show any significant differences. *n* = 5 mice per genotype. Values are mean ± SEM. **F** Representative Western blot and quantification of CD11b/c expression in hippocampus of nonTg+GFP, 3xTg+GFP and 3xTg+CPE mice at age ~8 months. CD11b/c was increased in 3xTg+GFP in comparison with nonTg+GFP, *p = 0.0175; but not significantly different from 3xTg+CPE. One-way ANOVA analysis followed by Tukey’s post-hoc multiple comparison test, [*F*_(2,12)_ = 5.401, *p* = 0.0212]. *n* = 5 mice per genotype. Values are mean ± SEM. **G** Representative sections of CD68 immunostaining for activated microglia in hippocampal CA1 of nonTg+GFP, 3xTg+GFP and 3xTg+CPE mice at age ~8 months. Magnification 20x, scale bar = 50 μm. *n* = 6 mice per genotype. **H** Quantification of CD68 positive cells in hippocampal CA1 of nonTg+GFP, 3xTg+GFP and 3xTg+CPE mice at age ~8 months. CD68 positive cells were significantly increased in 3xTg+GFP in comparison with nonTg+GFP, *p = 0.0016. Overexpression of NF-α1/CPE in 3xTg mice reduced activated microglia ^#^*p* = 0.0323. One-way ANOVA analysis followed by Tukey’s post-hoc multiple comparison test, [*F*_(2,15)_ = 9.728, *p* = 0.002]. 4 sections per mouse, *n* = 6 mice per genotype. Values are mean ± SEM. **I** Representative sections of doublecortin (DCX) stained immature neurons in subgranular zone of dentate gyrus of nonTg+GFP, 3xTg+GFP and 3xTg+CPE mice at age ~8 months. Magnification 20x, scale bar = 50 μm. *n* = 6 mice per genotype. **J** Quantification of DCX positive cells in subgranular zone of dentate gyrus of nonTg+GFP, 3xTg+GFP and 3xTg+CPE mice at age ~8 months. DCX positive cells were significantly decreased in 3xTg+GFP in comparison with nonTg+GFP, *p = 0.015. Overexpression of NF-α1/CPE in 3xTg+CPE did not mitigate this deficit in neurogenesis *p* = 0.9947. One-way ANOVA analysis followed by Tukey’s post-hoc multiple comparison test, [*F*_(2,15)_ = 7.063, *p* = 0.0069]. 4 sections per mouse, *n* = 6 mice per genotype. Values are mean ± SEM.

In Fig. 3A, hippocampal neurodegeneration was examined in 3xTg-AD mice with and without delivery of AAV-NF-α1/CPE, and in non-Tg mice. MAP2 immunofluorescence staining of neurites in the hippocampal CA1 region of 3xTg-GFP mice showed neurodegeneration compared to AAV- NF-α1/CPE treated (3xTg-CPE) and non-Tg-GFP mice (Fig. 3A, and insets). Quantification of MAP2 intensity in the CA1 hippocampus (Fig. 3B) confirmed significantly (22.87%) lower MAP2 immunostaining in 3xTg-GFP mice compared to non-Tg mice. In contrast, MAP2 immunostaining in those mice treated with AAV- NF-α1/CPE, (3xTg-CPE mice), showed only a 9.25% decrease relative to non-Tg mice. Our results indicate that NF-α1/CPE overexpression in the hippocampus significantly prevented neurodegeneration in 3xTg-AD mice.

The effect of hippocampal injection of AAV-NF-α1/CPE in 3xTg-AD mice on glial cells were examined. Morphological analysis indicated no difference in GFAP immunostaining and glial cell numbers in the hippocampal CA1 region with and without AAV-NF-α1/CPE delivery (Fig. 3C, D). Western blot analysis of the hippocampus also revealed no difference in GFAP protein levels between 3xTg-AD mice treated, or not treated with AAV-NF-α1/CPE, and non-Tg mice, indicating no changes in glial cell quantity (Fig. 3E). Western blot analysis of a marker for microglia, CD11b/c, indicate a significant increase in microglia in the hippocampus of 3xTg-AD (3xTg-GFP) mice compared to non-Tg mice (Fig. 3F); AAV- NF-α1/CPE treatment showed a trend of decrease of this marker in 3xTg-CPE mice compared to 3xTg-GFP mice, but this did not reach statistical significance. However, when reactive microglia were analyzed morphologically using the marker CD68 staining, there was a significant decrease in the number in 3xTg-CPE versus 3xTg-GFP mice (Fig. 3G, H). These results indicate that AAV- NF-α1/CPE treatment significantly prevented microglia activation in the 3xTg-AD mice.

Since NF-α1/CPE has been shown to have effects on neurogenesis [22], we examined doublecortin immunostaining in the dentate gyrus of 3xTg-AD mice. Figure 3I, J shows that there was a significant decrease in the number of doublecortin (DCX) positive cells in the dentate gyrus of 3xTg-AD mice compared to non-Tg mice. Delivery of AAV- NF-α1/CPE in the hippocampus did not rescue the decrease in neurogenesis in 3xTg-AD mice.

### AAV-NF-α1/CPE down-regulates hippocampal tau phosphorylation and APP/Aβ42 levels in 3xTg-AD mice

**Fig. 4:**
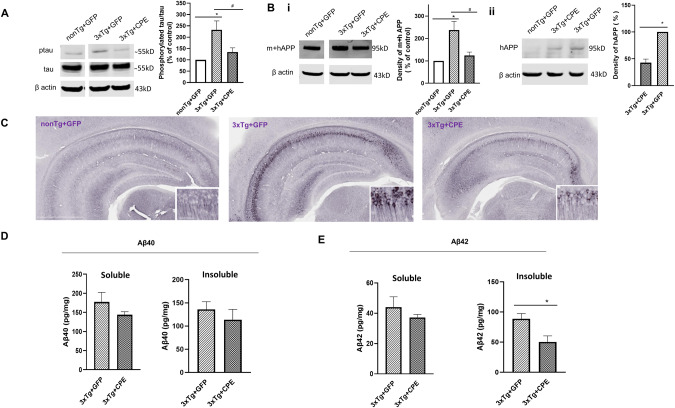
AAV-NF-α1/CPE reduces tau phosphorylation and APP and β-amyloid42 expression. **A** Representative Western blot and quantification of phosphorylated Tau expression in hippocampus of nonTg+GFP, 3xTg+GFP and 3xTg+CPE mice at age ~8 months. Phosphorylated tau at Ser396 was increased in hippocampus of 3xTg+GFP in comparison with nonTg+GFP, *p = 0.0075; overexpression of NF-α1/CPE in 3xTg+CPE mice significantly reduced phosphorylated tau, ^#^*p* = 0.0425. One-way ANOVA analysis followed by Tukey’s post-hoc multiple comparison test, [*F*_(2,12)_ = 7.497, *p* = 0.0077]. *n* = 5 mice per genotype. Values are mean ± SEM. **B** (i) Representative Western blot and quantification of mouse + human β-amyloid precursor protein (m + h APP) expression in hippocampus of nonTg+GFP, 3xTg+GFP and 3xTg+CPE mice at age ~8 months. β-amyloid precursor protein was significantly increased in 3xTg+GFP, *p = 0.0039; but decreased with overexpression of NF-α1/CPE in 3xTg+CPE mice, ^#^*p* = 0.0137. One-way ANOVA analysis followed by Tukey’s post-hoc multiple comparison test, [*F*_(2,12)_ = 9.622, *p* = 0.0032]. *n* = 5 mice per genotype. Values are mean ± SEM. (ii) Representative Western blot of human β-amyloid precursor protein (hAPP) expression in hippocampus of nonTg+GFP, 3xTg+GFP and 3xTg+CPE mice and quantification of hAPP of 3xTg+GFP and 3xTg+CPE at age ~8 months. β-amyloid precursor was decreased with overexpression of NF-α1/CPE in 3xTg+CPE mice, *p = 0.0001. *t* test, *n* = 3 mice per genotype. Values are mean ± SEM. **C** Representative image of β-amyloid precursor expression in hippocampus of nonTg+GFP, 3xTg+GFP and 3xTg+CPE mice at age of ~8 months. Magnification 2x, scale bar = 1 mm. Inset: β-amyloid precursor expression in CA1 area, magnification 20x, scale bar = 50 μm. **D** Human β-amyloid40 in hippocampus of 3xTg+GFP and 3xTg+CPE mice at age ~8 months. Both soluble and insoluble β-amyloid40 showed no significant difference between 3xTg +GFP versus 3xTg+CPE mice. Student *t* test. *n* = 5 mice per genotype. Values are the mean ± SEM. **E** Human β-amyloid42 in hippocampus of 3xTg+GFP and 3xTg+CPE mice at age ~8 months. Soluble β-amyloid42 showed no significant difference between 3xTg+GFP and 3xTg+CPE. Insoluble β-amyloid42 was decreased in 3xTg+CPE compared to 3xTg+GFP mice, Student *t* test, *p = 0.0194, *n* = 5 mice per genotype. Values are the mean ± SEM.

Hyperphosphorylation of tau leading to neurofibrillary formation is a characteristic pathology of AD. Figure 4A and Supplementary Fig. S5A show increased tau phosphorylation in the 3xTg-AD compared to non-Tg mice. Hippocampal delivery of AAV-NF-α1/CPE significantly decreased tau hyperphosphorylation in these mice (3xTg-CPE) to levels comparable to those in non-Tg mice.

Since increased amyloid Aβ42 production and deposition is a hallmark of AD, we examined the expression of APP and Aβ42 levels in 3xTg-AD mice with and without AAV-NF-α1/CPE treatment. Western blot analysis showed highly elevated levels of human+mouse (h+m)APP expression in 3xTg-AD mice compared to mAPP in non-Tg mice. The h+mAPP in 3xTg-AD mice was significantly attenuated to a level similar to non-Tg mice after AAV-NF-α1/CPE treatment (Fig. 4Bi). Specific analysis of hAPP protein level in 3xTg-AD mice showed significant decrease with AAV-NF-α1/CPE treatment (Fig. 4Bii). Morphological studies showed strong APP specific immunostaining in the CA1–3 regions of the hippocampus, and localized in the cell body and neurites of the neurons in 3xTg-AD mice (Fig. 4C middle panel). However, in 3xTg-CPE mice, (Fig. 4C right panel), APP immunostaining was greatly attenuated to levels comparable to non-Tg mice (Fig. 4C left panel). Thus AAV-NF-α1/CPE treatment greatly decreased the number of APP positive cells in the hippocampal CA1-3 regions in 3xTg-CPE mice compared to 3xTg-GFP mice.

Analysis of hAPP processed products showed only insoluble Aβ42(Fig. 4E), but not soluble or insoluble Aβ40 (Fig. 4D) was significantly decreased in 3xTg-CPE mice in comparision with 3xTg-GFP. This result suggests that AAV-NF-α1/CPE treatment of 3xTg-AD mice inhibited the up-regulation of APP expression and significantly decreased insoluble Aβ42 production in these mice.

### AAV-NF-α1/CPE gene delivery promotes mitochondrial function in 3xTg-AD mice

**Fig. 5:**
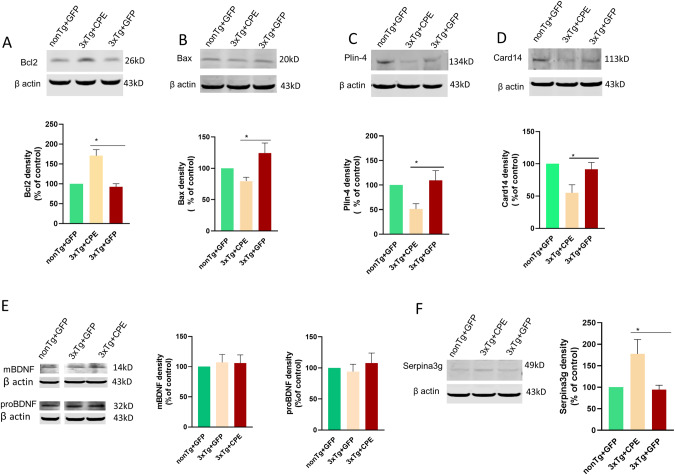
AAV-NF-α1/CPE gene delivery on hippocampal Bcl2, Bax, Plin4, Card14, BDNF and Serpina3g expression in 3xTg-AD mice. **A** Representative Western blot and bar graph showing Bcl2 was increased in hippocampus of 3xTg+CPE in comparison with 3xTg+GFP, *p = 0.0003. One-way ANOVA analysis followed by Tukey’s post-hoc multiple comparison test, [*F*_(2,12)_ = 19.22, *p* = 0.0002]. *n* = 5 per genotype. Values are the mean ± SEM. **B** Representative Western blot and bar graph showing Bax was decreased in the hippocampus of 3xTg+CPE in comparison with 3xTg+GFP, *p = 0.0179. One-way ANOVA analysis followed by Tukey’s post-hoc multiple comparison test, [*F*_(2,12)_ = 5.275, *p* = 0.0227]. *n* = 5 per genotype. Values are the mean ± SEM. **C** Representative Western blot and bar graph showing Plin4 was decreased in the hippocampus of 3xTg+CPE in comparison with 3xTg+GFP mice, *p = 0.0219. One-way ANOVA analysis followed by Tukey’s post-hoc multiple comparison test, [*F*_(2,12)_ = 5.655, *p* = 0.0186]. *n* = 5 per genotype. The values are the mean ± SEM. **D** Representative Western blot and bar graph showing Card14 was decreased in the hippocampus of 3xTg+CPE in comparison with 3xTg+GFP mice, *p = 0.045. One-way ANOVA analysis followed by Tukey’s post-hoc multiple comparison test, [*F*_(2,12)_ = 6.391, *p* = 0.0129]. *n* = 5 per genotype. The values are the mean ± SEM. **E** Representative Western blot and bar graph showing quantification of pro-BDNF and mature (m) BDNF expression in the hippocampus of nonTg+GFP, 3xTg+GFP and 3xTg+CPE mice. No significant differences were observed in mBDNF and proBDNF among the three groups. One-way ANOVA analysis followed by Tukey’s post-hoc multiple comparison test. *n* = 4 per genotype. The values are the mean ± SEM. **F** Representative Western blot and bar graph showing Serpina3g protein was increased in the hippocampus of 3xTg+CPE in comparison with 3xTg+GFP mice. *p = 0.032. One-way ANOVA analysis followed by Tukey’s post-hoc multiple comparison test, [*F*_(2,12)_ = 5.315, *p* = 0.0222]. *n* = 5 per genotype. The values are the mean ± SEM.

Since NF-α1/CPE is known to regulate mitochondrial pro-survival and energy metabolism genes [22, 23], we examined the effect of AAV-NF-α1/CPE treatment on expression of mRNA and proteins encoded by some of these genes. Expression of Bcl2, a pro-survival protein (Fig. 5A) was found to be significantly increased, while Bax, a pro-apoptotic protein was decreased in 3xTg-CPE compared to 3xTg-GFP mice (Fig. 5B). The Bcl2/Bax ratio was 2.14 and 0.74 for 3xTg-CPE and 3xTg-GFP, respectively, indicating that hippocampal AAV-NF-α1/CPE treatment promotes cell survival in 3xTg-AD mice through modulating expression of these two mitochondrial membrane proteins [33].

AD subjects show low glucose metabolism in the brain which seems to be compensated by shifting to amino acids and fatty acids as alternative energy sources [34, 35] NF-α1/CPE is known to play a role in energy metabolism in pre-osteoblastsic cells where it increased mitochondrial oxidative phosphorylation and cellular dependence on fatty acid for energy production, in lieu of glycolysis [23]. We analyzed the expression of three gene transcripts *ND1*, (encoding NADH dehydrogenase subunit 1), *CO1 and CO3* (encoding cytochrome c oxidase subunit 1 and 3, respectively), as well as several mitochondria respiratory chain subunits (CI-NDUFB8, CII-SDHB, CIV-MTCO1, CV-ATP5A) in 3x Tg-AD mice (Supplementary Figs. S3 and S4). No difference was observed between 3xTg-GFP, 3xTg-CPE and non-Tg mice, suggesting that energy metabolism gene expression is not significantly altered in 3xTg-AD mice at ~8 months of age.

Studies have revealed that phosphorylated Tau and Aβ induce defective mitophagy in AD, an important process that removes dysfunctional mitochondria in neurodegenerative diseases [36]. *Plin4* is a gene that encodes a protein that inhibits mitophagy [37] and is increased in an induced AD model [38]. We therefore investigated the expression of this mitophagy inhibitor. We showed that 3xTg-GFP mice had similar levels of plin4 protein as in non-Tg mice, but 3xTg -CPE mice had significantly decreased levels of this protein (Fig. 5C). This result indicates that AAV- NF-α1/CPE gene delivery may facilitate mitophagy in AD mice through reduction of Plin4 expression.

### Effect of hippocampal AAV-NF-α1/CPE injection on Card14, BDNF and serpina3g expression

In AD, it has become increasingly clear that neuroinflammation mediated by reactive astrocytes and microglia contributes to the pathogenesis of the disease [39]. We have examined the effect of AAV- NF-α1/CPE gene delivery into the hippocampus of 3xTg-AD mice on the expression of the neuroinflammation-related protein, Card14 found in microglia [37, 40]. Card14 protein was significantly reduced in the 3xTg-CPE vs 3xTg-GFP mice (Fig. 5D). Decreased levels of Card14 after AAV-NF-α1/CPE treatment in 3xTg-CPE mice may serve to mitigate neuroinflammation in these mice.

BDNF, an important regulator of synaptic plasticity and neuronal survival, has been shown to alleviate cognitive dysfunction in AD mouse models upon delivery of BDNF gene to the hippocampus [19, 41]. Hippocampal delivery of AAV-NF-α1/CPE had no effect on increasing pro-BDNF and BDNF protein levels in 3xTg-AD mice (Fig. 5E) and therefore BDNF is unlikely to account for the improvement of cognitive function in the 3xTg-CPE mice. Furthermore, there was no difference in pro- or mature BDNF levels between 3xTg-GFP and non-Tg mice at this age, indicating that the AD pathology observed is not due to a deficiency of BDNF.

**Fig. 6:**
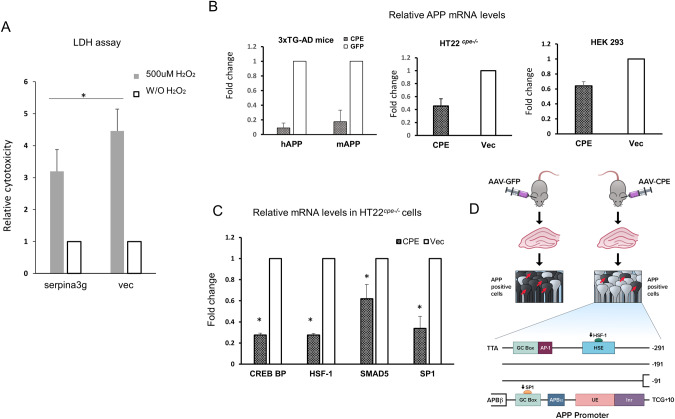
Factors regulating neuronal survival and *APP* mRNA transcription induced by NF-α1/CPE. **A** Serpina3g protects HT22 hippocampal neurons against H_2_O_2_-induced cytotoxicity. Bar graphs (stippled bars) showing that cell cytotoxicity decreased in HT22 cells expressing serpina3g compared to cells expressing vector alone. Open bars showing cytotoxicity level (made = 1) in the control cells expressing serpina3g or vector, but not treated with H_2_O_2_. Values shown are the mean ± SEM, *p* < 0.05, *t*-test, *N* = 3. **B** Reduction of *APP* mRNA levels in 3xTg-AD mice and cells after NF-α1/CPE gene delivery. Left panel: Bar graphs showing relative human and mouse *APP* mRNA levels in 3xTg-AD mouse hippocampus after delivery of AAV-NF-α1/CPE or AAV-GFP. Values shown are the mean ± SD, *n* = 2 mice per genotype. Middle panel: HT22^*cpe−/−*^ cells were transiently transfected with CPE or vector for 48 h and then harvested for qRT- PCR assay. Bar graphs showing relative *mAPP* mRNA level. Values shown are the mean ± SEM, *N* = 3. Right panel: HEK293 cells were transiently transfected with CPE and vector for 48 h and then harvested for qRT- PCR assay. Bar graphs showing relative *hAPP* mRNA level. Values shown are the mean ± SEM, *N* = 3. **C**. Reduction of transcription factor mRNAs in HT22^*cpe−/−*^ cells after CPE transfection. HT22^*cpe−/−*^ cells were transiently transfected with CPE or vector for 48 h and then harvested for qRT- PCR assay. Bar graphs showing relative mRNA levels of 4 transcription factors were reduced with CPE transfection. Values shown are the mean ± SEM, *N* = 3. *p < 0.05, **p < 0.01, ***p < 0.001. **D** Schematic showing hippocampal injection of AAV- NF-α1/CPE in 3xTg-AD mice down-regulates expression of APP in pyramidal neurons via attenuating transcription factors Sp1 and Hsf-1 expression. These transcription factors bind to the promoter of *APP* gene to promote transcription. Red arrows indicate APP positive neurons.

Murine Serpina3g (α1-anti-chymotrypsin) is one of the mouse orthologues to human SERPINA3 [42], a microglia protein that is overexpressed in brain of AD patients [42]. Serpina3g shares 55% amino acid homology with SERPINA3 [42], and is expressed in reactive astrocytes, microglia and neurons (our unpublished data). It has been reported to have pro-survival activity [43]. We found that after delivery of AAV-NF-α1/CPE into hippocampus of 3xTg CPE mice, serpina3g protein level was significantly elevated by ~180% compared to 3xTg-GFP mice (Fig. 5F). Serpina3g level was similar between 3xTg-GFP and non-Tg mice. We then investigated whether serpina3g could have pro-survival activity in neurons. *Serpina3g* gene transfected into HT22 cells, a model mouse hippocampal cell line increased *serpina3g* mRNA expression 9477 ± 986 fold (*p* < 0.05, *N* = 3). These neurons were then challenged with H_2_O_2_ to induce oxidative stress and assayed for cell survival. Figure 6A shows that HT22 cells transfected with *serpinin3g* gene were protected from H_2_O_2_ -induced cytotoxicity, compared to cells transfected with vector alone. This finding indicates that serpinin3g can function as a neuroprotective, pro-survival protein. Thus the increase in serpina3g expression with AAV-NF-α1/CPE treatment may serve to promote neuronal survival in 3xTg-CPE mice.

### NF-α1/CPE down-regulates APP transcription via attenuation of Sp1 and Hsf-1 transcription factor expression

Expression of human (transgene under the Thy-1 promoter) and mouse APP were both down-regulated at the mRNA level (Fig. 6B left panel) in 3xTg-AD mice treated with AAV-NF-α1/CPE. To explore the mechanism involved in regulating transcription of APP by NF-α1/CPE, two models cell lines, mouse hippocampal HT22^*cpe*−/−^ and human HEK293 cells were transfected with the CPE gene. Figure 6B shows that NF-α1/CPE down-regulated expression of *APP* mRNA in both HT22^*cpe*−/−^ (middle panel) and HEK293 (right panel) cells. We then carried out a transcription signaling pathway microarray screen, (available only for human), on HEK293 cells transfected with NF-α1/CPE. Array analysis revealed that two transcription factors *HSF-1* and *SP1*, as well as *CREBBP* and *SMAD5* transcripts were down-regulated in NF-α1/CPE transfected HEK293 cells (Supplementary Table S3). We then showed that these factors were also down-regulated in HT22^*cpe−/−*^ cells transfected with NF-α1/CPE compared to mock transfected cells (Fig. 6C). These results suggest that NF-α1/CPE suppresses mouse *APP* mRNA expression in hippocampal neurons of 3xTg-AD mice through down-regulating transcription factors *Sp1 and Hsf-1* expression. These two transcription factors are known to bind to the mouse APP promoter to up-regulate transcription [44] (Fig. 6D). *Crebbp* and *Smad5* transcripts were also down-regulated in NF-α1/CPE transfected HT22^*cpe*−/−^ cells. Since SP1 is also a transcription factor for Thy-1 promoter, the down-regulation of human APP in 3xTg-AD mice with AAV-NF-α1/CPE treatment may be mediated by a decrease of this transcription factor.

## Discussion

AD is driven by multifactorial processes. The causes are not limited to amyloid and tau pathology, but also mitochondrial dysfunction and neuroinflammation, leading eventually to neuronal cell death [45]. This renders the search for therapeutic agents much more challenging. Current therapeutic agents targeting one of these pathologies at a time, e.g. amyloid clearance, have resulted in temporary improvements of memory loss in some cases [9, 12–14]. Gene therapy approaches with different growth factors have yielded some success in reversing cognitive dysfunction in animal models, independent of amyloid, but clinical trials have failed, although a trial with BDNF is still ongoing [20]. Thus far, there is no therapy that will prevent progression or reverse AD.

Our preclinical studies indicate that bilateral hippocampal delivery of AAV-NF-α1/CPE in pre-symptomatic male 3xTg-AD mice prevents progression of AD later in life, including rescuing cognitive dysfunction, tau hyperphosphorylation and APP/ Aβ42 overexpression. AAV-NF-α1/CPE gene delivery also promoted mitochondrial function and cell survival. 3xTg-AD mice express NF-α1/CPE in amounts comparable to WT and non-Tg mice in the hippocampus. Bilateral injection of AAV-NF-α1/CPE into the hippocampus increased expression of NF-α1/CPE protein by ~1.7 fold relative to 3xTg-GFP which was maintained for at least 6 months, and the anatomical pattern of expression of NF-α1/CPE was similar to control GFP injected mice. AAV gene delivery can maintain expression levels for more than a year [46]. In our experimental paradigm, all 3xTg-AD mice were injected with AAV-NF-α1/CPE at 2 months of age when they are pre-symptomatic [32] and assayed for cognitive function, morphological and biochemical changes in the hippocampus at age 7–8 months when AD pathology is evident. AAV-NF-α1/CPE treated mice showed no spatial memory deficit at 7 months of age in the Morris Water Maze tests, unlike their littermates injected with AAV-GFP. In addition, the Novel Object Recognition test showed a trend suggesting that AAV-NF-α1/CPE treatment improved memory in the 3xTg-AD mice. Thus NF-α1/CPE overexpression prevented the development of cognitive dysfunction found in 3xTg-AD mice. This is consistent with MAP2 immunostaining of neurites in the CA1 region showing that AAV-NF-α1/CPE treatment rescued neurodegeneration in the 3xTg-AD mice.

Biochemical analysis of the hippocampus showed that AAV-NF-α1/CPE treatment prevented hyperphosphorylation of tau at specific residues, Ser396, Ser202 and Thr205, known to be involved in AD [47–50]. Furthermore, the elevated APP found in 3xTg-AD mice was prevented in AAV-NF-α1/CPE treated mice, which had levels comparable to non-Tg mice. Immunocytochemical analysis indicate that the APP was localized mainly in the neuronal cell body in the 3xTg-AD mice. A recent report indicates that in AD mouse models, APP builds up in autophagic vacuoles around the nucleus which can then lead to cell death [51]. Additionally, insoluble toxic form of Aβ42 was significantly decreased in AAV-NF-α1/CPE treated 3xTg-AD treated mice, while the soluble form tended to be lower compared to untreated mice. Thus, the finding that AAV-NF-α1/CPE gene therapy can down-regulate APP expression in an AD mouse model is novel and represents an important new tool in AD treatment. BDNF [19], NGF [18] and FGF2 [21] gene therapy did not have such effects on alleviating tau or APP/Aβ42 pathology in their respective pre- or post-symptomatic AD mouse models.

To understand the signaling mechanism underlying the suppression of mouse *APP* (*mAPP*) mRNA expression by NF-α1/CPE at the transcriptional level, we used a mouse hippocampal cell line HT22^*cpe−/−*^. Transfection of these cells with NF-α1/CPE down-regulated the expression of *APP* along with *Hsf-1, Sp1, Crebbp and Smad5. Sp1* and *Hsf-1* mRNA which are transcription factors that bind to specific sites in the APP promoter to up-regulate expression [44] (see Fig. 6D). Their decreased expression would provide a mechanism for the attenuation of *mAPP* mRNA expression in hippocampal neurons in 3xTg-AD mice. Consistent with this mechanism is the finding that Sp1 is co-localized specifically with APP expressing pyramidal cells in the hippocampal CA1-3 regions [52]. The down-regulation of transcriptional co-activators CREBBP (or CBP), and SMAD5, with NF-α1/CPE transfection in hippocampal cells is interesting and suggest that they may participate in preventing NF-α1/CPE mediated AD pathology. However, understanding the physiological significance of the attenuation of these 2 transcriptional co-activators by NF-α1/CPE in AD will require further study.

In this study, we found that serpina 3g, (α1-antichymotrypsin) expression was up-regulated upon injection of AAV-NF-α1/CPE in the hippocampus of 3xTg-AD mice. We then demonstrated that serpina3g has neuroprotective activity against oxidative stress in hippocampal neurons. This is consistent with the pro-survival activity of serpina3g reported in erythroblasts [43]. Thus, serpina3g may be playing a neuroprotective role to prevent AD progression in 3xTg-AD mice. The human homolog of serpina3g is SERPINA3, (α1-antichymotrypsin) [53] and it is elevated in plasma and cerebrospinal fluid (CSF) of AD patients relative to healthy controls [54]. Interestingly, in CSF, a significant negative correlation was found between SERPINA3 and cognitive impairment based on Mini-mental state examination (MMSE) score [54]. SERPINA3 is highly secreted from reactive astrocytes and found in amyloid plaques [55], as well as many types of tumors including glioblastoma, and shown to promote proliferation and survival of cancer cells [56]. Whether SERPINA3 could play a compensatory neuroprotective role in AD progression in humans, similar to serpina3g, remains to be determined. Indeed, another serpin, neuroserpin, has been shown to have neuroprotective activity against oxidative stress in neurons [57].

Hippocampal AAV-NF-α1/CPE treatment down-regulated the expression of Card14 in 3xTg-AD mice. Card14 is expressed primarily in oligodendrocytes and microglia cells and less in neurons [58]. It recruits interacting partners BCL_10_ and MALT_1_ forming a complex that initiates NFκB pathway leading to up-regulation of expression of pro-inflammatory genes [40]. Indeed, while GFAP immunostaining revealed no difference in glial cell numbers between 3xTg-GFP, 3xTg-CPE and non-Tg mice, CD11b/c, the microglia marker was significantly increased in the 3xTg-AD compared to non-Tg mice, indicative of neuroinflammation. AAV-NF-α1/CPE treatment of 3xTg-AD mice resulted in a downward trend in expression of this marker. Moreover, immunostaining for the microglia marker, CD68, confirmed a significant decrease in the number of reactive microglia in NF-α1/CPE treated 3xTg-CPE compared to 3xTg-GFP mice. Down-regulation of expression of Card14, may contribute to the mitigation of neuroinflammation by microglial cells in the AAV-NF-α1/CPE treated 3xTg-AD mice. The mechanism underlying the decrease in reactive microglia numbers in treated 3xTg-AD mice may not be entirely due to a direct effect of overexpression of NF-α1/CPE in microglia, since transduction efficiency of transgenes into these cells is reported to be ≤20% using AAV injected into the brain in vivo [59]. Indirect effects such as reduction of amyloid load, or increased NF-α1/CPE secretion from neurons and asytocytes that might impact microglia, or perhaps affect secretion of agents (e.g. chemokines/cytokines, neurotramsmitters), thought to be important for crosstalk with microglia, could also contribute to reduced microglia inflammation.

Mitochondial dysfunction and pathogenesis are evident in AD brains resulting in ROS damage during oxidative stress [58, 60]. AAV-NF-α1/CPE treatment in 3xTg-AD mice effectively increased mitochondrial Bcl2, a pro-survival protein, and down regulated Bax expression, the pro-apoptotic protein that induces cytochome c leakage in the absence of inhibition by Bcl2, leading to cell death [33]. These findings show that NF-α1/CPE protects neurons against oxidative stress or other stress-mediated cell death in 3xTg-AD mice, via increasing mitochondrial Bcl2 and decreasing Bax expression, consistent with numerous studies demonstrating such neuroprotective activity of CPE [61, 62].

Dysfuctional mitochondria prevalent in neurodegenerative diseases, including AD, undergo mitophagy, a quality control process which selectively degrades mitochondria by autophagy following damage [63]. Mitophagy is inhibited by perilipin 4 (Plin4) and genetic silencing of *plin 4* reversed MPP + -induced mitochondria damage in SHSY5Ycells, a dopaminergic cell line [37]. Moreover, *plin4* is up-regulated in sevoflurane-induced AD-related neuropathalogy and cognitive dysfunction in mice, presumably causing inhibition of mitophagy [38]. Thus the down-regulation of expression of Plin4 protein with AAV-NF-α1/CPE treatment in 3xTg-AD mice renders another mechanism for promoting neuronal survival in these mice through activating mitophagy, an important “housekeeping” process to degrade damaged mitochondria in AD.

In conclusion, AAV-NF-α1/CPE gene delivery at an early age in the hippocampus prevented subsequent progression of AD in male 3xTg-AD mouse model via targeting various regulatory components to restore homeostasis. Most importantly hippocampal AAV-NF-α1/CPE treatment promoted mitochondrial Bcl2-, and serpina3g- mediated cell survival mechanisms, down-regulated APP transcription and reduced insoluble Aβ42 production and tau hyperphosphorylation to alleviate cognitive dysfunction in 3xTg-AD mice. Additionally, AAV-NF-α1/CPE overexpression suppressed Plin4 expression thereby activating mitophagy, and decreased a microglial pro-inflammatory protein, Card14. NF-α1/CPE is the first trophic factor shown to be able to rescue multiple key pathologies associated with AD and stop progression of the disease. Future studies are warranted to determine if NF-α1/CPE treatment will have the same effects on female 3xTg-AD mice. Notably, AAV-NF-α1/CPE treatment in hippocampus has no obvious adverse effect on non-Tg mice (Supplementary Fig. S5B–F). Furthermore, the effect of NF-α1/CPE in preventing memory loss in 3xTg-AD mice does not depend on its enzymatic activity (our unpublished data). Thus, our findings in 3xTg-AD mice have implications for AAV-NF-α1/CPE gene therapy as a potentially promising approach to treating AD in humans, especially in familial AD and early disease stage patients.

## Material and methods

### Animals

Male wild type, (C57BK/6J), 3xTg-AD (Cat# 004807) and nonTg mice (Cat# 101045) were purchased from Jackson Labs (Bar Harbor, ME). All mice were housed at NIH animal facility with free access to food and water ad libitum and controlled humidity (45%) and temperature (22 °C) under a 12 h light/dark cycle. All animal study protocols were approved by the Animal Care and Use Committee of NICHD, NIH and complied with ethical regulations. At age of 2 months, 3xTg and nonTg mice were randomly allocated into 4 groups: 3xTg+GFP, 3xTg+CPE, nonTg+GFP and nonTg+CPE to receive bilateral hippocampal stereotaxic injections. At age of ~8 months, brain tissues were collected for biochemical and immunohistochemical studies after behavioral tests carried out at 7 months of age.

### Viral vectors and plasmid constructs

AAV1/2-GFP and AAV1/2-CPE (chimeric serotype) viral constructs were purchased from Vector Biolabs (Philadelphia, PA). AAV1 and AAV2 both transduce neurons, astrocytes and reactive glia. GFP and CPE expression in these AAV constructs are driven by the CMV promoter. Plasmids expressing human or mouse CPE carrying a C-terminal V5 tag were constructed into pcDNA3.1 backbone by Genscript (Piscataway, NJ). Plasmids expressing mouse serpina3g was from Origene Technologies (Rockville, MD, Cat # MR216778).

### Cells

HEK293 cells were purchased from ATCC (Manassas, VA). HT22, a mouse hippocampal cell line was obtained from Salk Institute (La Jolla, CA). CPE knockout HT22 cells (HT22^cpe−/−^) were generated by CRISPR-Cas9 method as previously described [24].

### Stereotaxic injection

Mice were prepared and immobilized in stereotaxic apparatus according to protocol. AAV viruses expressing mouse CPE or GFP were bilaterally injected into the hippocampus (total 1 × 10^10^ VP,1ul on each side of hippocampus) according to the coordinates AP: −1.94 mm, L: ±1.0 mm, V: −1.3 mm. (injection site is shown in Supplementary Fig. S1A). Injection controls were performed on 3xTg-AD mice; no difference in APP or ptau expression in hippocampus with and without injection was observed (Supplementary Fig. S1B, C).

### Behavioral studies

NonTg and 3xTg-AD mice at 2 months of age were injected with either AAV-GFP or AAV-NF-α1/CPE in the hippocampus, and at ~8 months of age, they were subjected to a series of behavioral studies as described in Supplementary Method, in the order of open field, the novel object recognition, elevated plus maze, Morris water maze, and forced-swim test.

### ELISA for Aβ40 and Aβ42

Extraction of soluble and insoluble proteins was performed as previously described [32]. Hippocampal tissues homogenates were centrifuged at 4 °C for 30 min at 100,000 g. The supernatant containing soluble proteins was removed and saved. The precipitate containing insoluble proteins was resuspended in 70% formic acid (Sigma-Aldrich, St. Louis, MO). Both the supernatant and precipitate were used for ELISA analysis. Soluble and insoluble Aβ40 and Aβ42 were detected with ELISA kit specific for human, following manufacture’s instructions (Invitrogen, Waltham, MA).

### Western blot

Mouse brain tissues were prepared in RIPA (Thermofisher, Waltham, MA) supplemented with 1× Complete Inhibitor Cocktail (Roche,) and centrifuged. Lysates were used for Western blot and run on SDS-PAGE gels and transferred onto nitrocellulose membrane. The membrane was incubated with primary antibodies (see Supplementary Table S1), overnight after blocking with 5% nonfat milk for 1 h, and then with secondary fluorescent conjugated anti-mouse or rabbit antibodies. The bands were visualized and quantified by the Odyssey infrared imaging system (LI-COR Inc, Lincoln, NE). The protein expression level for each sample was normalized to β-actin.

### Immunohistochemistry

Mouse brains were sectioned coronally at 25 µm and sections were stained with primary antibodies (See Supplementary Table S2) and then followed by 1:1000 biotinylated (Vector, Newark, CA) or 1:500 fluorescence (Jackson Lab, Bar Harbor, ME) secondary antibodies. Images were obtained with an Olympus VS200 slide scanning system. To quantify newborn neurons, DCX positive cells were counted in the dentate gyrus at 20× (four sections per animal, six animals per genotype). To quantify MAP2 staining in CA1 region, two random areas (171 × 171 µm) at 10× (in each of four sections per animal, six animals per genotype) were selected and intensity was measured by Image J. To quantify GFAP positive cells in CA1 region, two random areas (171 × 171 µm) at 10× (in each of four sections per animal, six animals per genotype) were selected and positive cells in those areas were counted. To quantify CD68 positive cells, numbers of positive cells and total cells in CA1 region were counted within a 260 μm × 320 μm area (4 sections per mouse, 6 mice per genotype), percentage of positive cells versus total cell numbers was calculated.

### Quantitative real-time PCR (qPCR)

Total RNA was isolated from mouse hippocampus or cells using trizol method and reverse transcribed using SensiFAST cDNA Synthesis Kit (cat. BIO-65054). Details of the gene specific primers are given in Supplementary Tables S4 and S5. Forty PCR amplification cycles were performed with the following protocol: 50 °C for 2 min, 95 °C for 10 min, 95 °C for 15 s, 60 °C for 1 min in a Quant Studio (TM) 6 Flex PCR using SYBER green master mix (Applied Biosystems, Foster City, CA, USA). For the expression analysis of mitochondrial metabolism genes ND1, CO-1 and CO-3, TaqMan™ Gene Expression Assays (BioRad USA) and Fast Advanced Master Mix (Cat. no. 4444556, Applied Biosystem, USA) were used. Threshold values (Ct) were determined automatically by the Quant Studio Real-Time PCR Software and relative mRNA expression was analyzed by the comparative ΔΔCt method. Data were normalized to 18S or GAPDH. Primer sequences used are reported in Supplementary. Specific primers were designed for human APP mRNA and mouse APP mRNA.

### Lactate dehydrogenase (LDH) cytotoxicity assay of HT22 cells with serpina3g transfection

The LDH assay was carried out using a CytoTox 96® Non-Radioactive Cytotoxicity Assay kit (Promega, Cat#) to measure release of LDH from damaged cells. Briefly, 1 × 10^6^ of HT22 cells were seeded into 6-well plate overnight and the next day, 4 ug of Serpina3g or pcDNA3.1 empty plasmid were transfected into the cells using Lipofectamine 2000 (Thermo Fisher, Cat#11668019) according to manufacturer’s instructions. Forty eight hours after transfection, 1 × 10^4^ transfected cells were reseeded into 96 well plate overnight. The following day, regular DMEM medium was removed, and cells were treated with 200 µl of serum free DMEM medium containing 500 uM H_2_O_2_ for 4 h, then 50 µl of the medium supernatant was used for LDH assay. Cytotoxicity was calculated relative to control cells.

### TaqMan Human Transcription Factors array

TaqMan Human Transcription Factors array (Cat# 4418784, Thermo Fisher) was used to assess different transcription factors as described in Supplementary Method and Supplementary Table S3 for details.

### Statistical analysis

Data are representative of at least 3 separate experiments (N), and each experiment was done in triplicates (*n* = 3) unless specified otherwise in text. Data were analyzed by 2-tail Student’s *t* test or 1-way ANOVA, or 2-way ANOVA followed by Tukey’s post hoc multiple comparisons tests, where noted. Analysis was performed with assistance of GraphPad Prism (GraphPad, La Jolla, CA) software package. Significance was set at *p* < 0.05.

### Supplementary information

Supplementary Information

## Data Availability

The data that were used to support the findings of this study are available from the corresponding author upon reasonable request.
