# Correction: Hippocampal delivery of neurotrophic factor-α1/carboxypeptidase E gene prevents neurodegeneration, amyloidosis, memory loss in Alzheimer’s Disease male mice

**DOI:** 10.1038/s41380-024-02633-2

**Published:** 2024-06-08

**Authors:** Lan Xiao, Xuyu Yang, Vinay Kumar Sharma, Daniel Abebe, Y. Peng Loh

**Affiliations:** Section on Cellular Neurobiology, Eunice Kennedy Shriver National Institute of Child Health and Human Development, National Institutes of Health, Bethesda, Md 20892 USA

**Keywords:** Neuroscience, Physiology

Correction to: *Molecular Psychiatry* 10.1038/s41380-023-02135-7, published online 28 June 2023

In this article the wrong figure appeared as Fig. 3; the figure should have appeared as shown below.
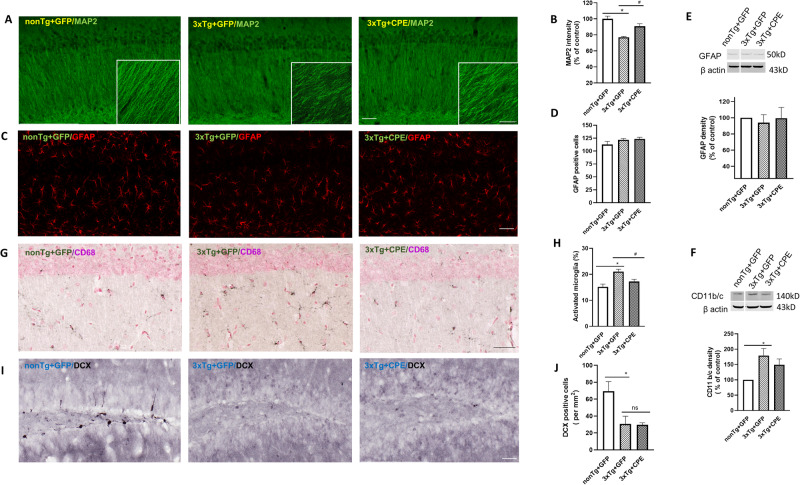


The original article has been corrected.

