# Supplementary material for: Hippocampal delivery of neurotrophic factor-α1/carboxypeptidase E gene prevents neurodegeneration, amyloidosis, memory loss in Alzheimer’s Disease male mice
Source: Mol Psychiatry. 2023 Jun 28;28(8):3332–42. doi: 10.1038/s41380-023-02135-7 (PMC10618095; doi:10.1038/s41380-023-02135-7)
Supplement: Supplementary file 1 — Supplementary Information [file 41380_2023_2135_MOESM1_ESM.docx]

**Supplementary Method**

**Behavioral studies**

*Open field test*

To evaluate the locomotor activity, mice were placed into the open field for 1 h. Moving distance and speed were monitored and analyzed by ANY-maze software (ANY-maze, Wood Dale, IL).

*The novel object recognition (NOR) test*

NOR test consists of three phases: on day 1, mice were habituated to the experimental arena in absence of objects for 10min; on day 2, mice were trained twice by being placed in the experimental arena with 2 objects and were allowed to explore for 10 minutes. On day 3, long-term memory was tested 24 hours after training. Mice were allowed to explore the experimental arena for 10 minutes in the presence of 1 familiar and 1 novel object. The novel objects were counterbalanced in all experiments and the objects and apparatus were cleaned by 70% ethanol between trials to avoid olfactory cues. Mice that spent less than 10 seconds on both familiar object and novel object on day 3 were excluded. Mice were tracked by ANY-maze software (ANY-maze, Wood Dale, IL). Recognition index is defined as time exploring new object/(time exploring new object+time exploring familiar objects) x100 were calculated.

*Elevated plus maze test*

The elevated plus maze test was performed as previously described ^1^. The apparatus is made of polypropylene and comprises two open and two close arms that extended from a central platform. Mice were individually placed in the central area facing open arm and allowed to freely explore the apparatus for 5 min. The time a mouse spent in open and close arms and entries into each arm were video recorded and analyzed with ANY-maze software.

*Morris Water Maze test*

Morris water maze was conducted as previously described ^2^. The test consists of a 5-day hidden platform training and 1-day probe test. Test was performed in a circular pool filled with water and nontoxic white paint. Video tracking and navigational parameters were analyzed with Any Maze software (ANY-maze, Wood Dale, IL). For training from day 1 to day 5, there were four trials each day and mice were placed in a new quadrant on each trial. The hidden platform was put in the same position for all five trials. Mice would search for the platform for 1 min. If mice did not find the hidden platform, they were guided to the platform and allowed to sit on it for 30 second. Escape latency, the time for mice to find the hidden platform was recorded for five days. On day 6, the hidden platform was removed, and the mice were allowed to explore the pool for 1 min. The time mice spent in each quadrant was recorded and analyzed by ANY-maze software (ANY-maze, Wood Dale, IL).

*Forced-swim test*

The forced-swim test was used to evaluate depressive-like behavior and was conducted as previously described ^3^. The apparatus was a transparent cylinder container filled with water at 23 °C. Swimming behavior was recorded for 6 min and immobility in the last 4 min was analyzed. Immobility time is defined as the time that the animal spent floating or engaged in minimal activity to keep their heads above the water.

**TaqMan Human Transcription Factors array**

Briefly, 1x10^6^ of HEK293 cells were seeded into 6-well plate overnight and the next day 4µg of CPE or pcDNA3.1 empty plasmid were transfected into the cells using Lipofectamine 2000 (Thermo Fisher, Cat#11668019) according to manufacturer’s instructions, respectively. Cells were harvested 48 hours after transfection and total RNAs were made using RNAeasy kit (Qiagen, Cat#: 74004) and then converted into cDNA using SensiFAST™ cDNA Synthesis Kit (Bioline, Cat# BIO-65054) according to the manufacturer’s protocols. The cDNAs were mixed with TaqMan® Fast Universal PCR Master Mix (Thermo Fisher, Cat# [4444557](https://www.thermofisher.com/order/catalog/product/4444557)) and PCR assays were run in QuantStudio 6™ PCR system following the standard protocol as described in the Transcription Factors array’s manual (Pub. No. 4391139 Rev. E, Applied Biosystem). The relative quantification (ΔΔCt) method was used to analyze PCR data against housekeeping gene GAPDH. Four transcription factors including CREBBP, HSF-1, SMAD5 and SP1 were validated with HEK293 and HT22*^cpe-/-^* cells by qPCR.

**Supplementary Figures**

**Figure S1**

**
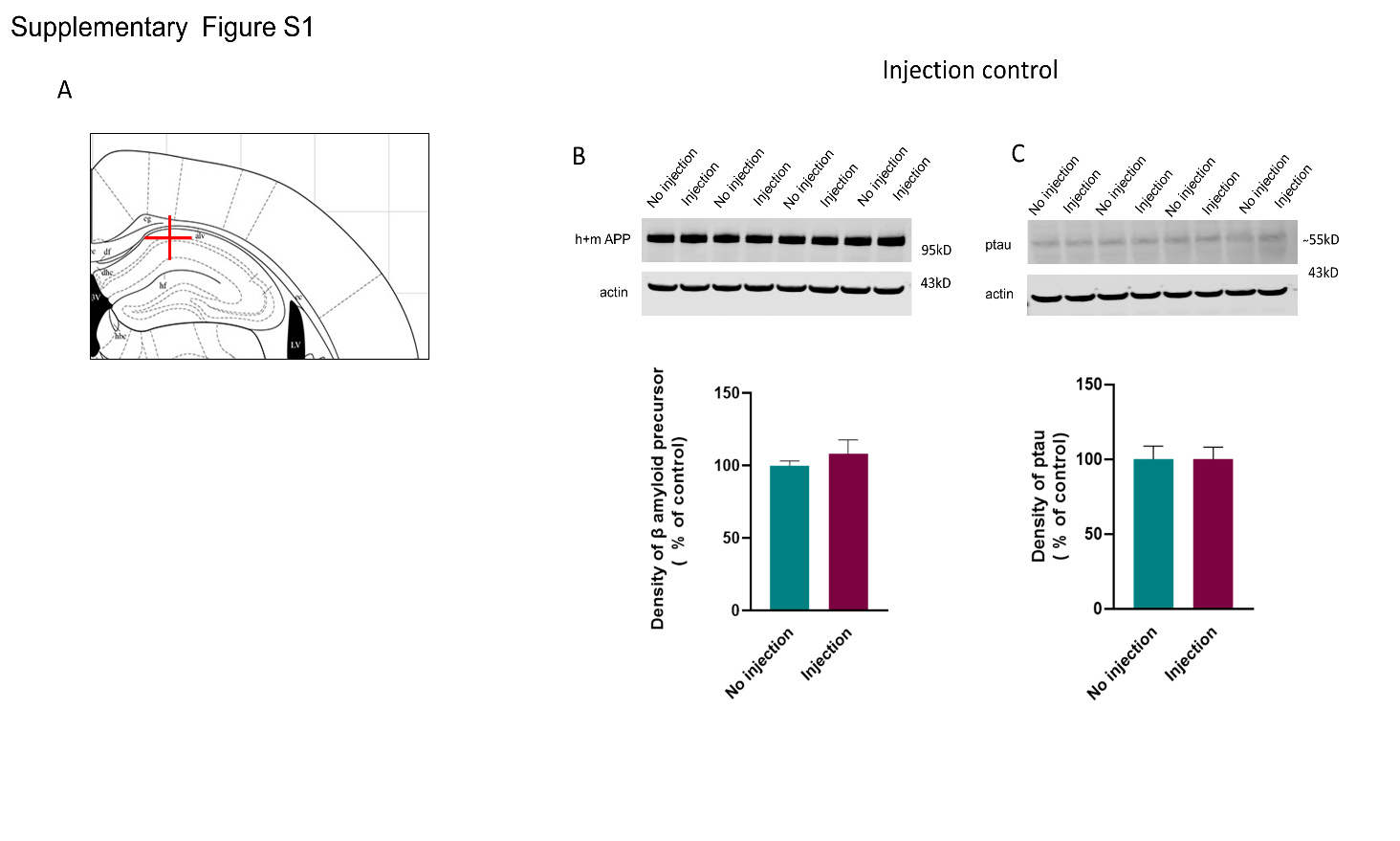
**

(A) Schematic diagram of injection site. Mice were bilaterally injected with AAV into the hippocampus according to the coordinates AP :-1.94mm, L: ± 1.0mm, V:-1.3mm.

(B) Representative Western blot and quantification of human+ mouse amyloid precursor protein (APP) expression in the hippocampus of 4 month old 3xTg-AD mice with AAV-GFP injection and uninjected mice. No significant differences were observed between two groups. t-test. n=4 per genotype. The values are the mean ± SEM.

(C) Representative Western blot and quantification of phosphorylated tau expression in the hippocampus of 4 month old 3xTg-AD mice with AAV-GFP injection and uninjected mice. No significant differences were observed between two groups. t-test. n=4 per genotype. The values are the mean ± SEM.

**Figure S2**

**
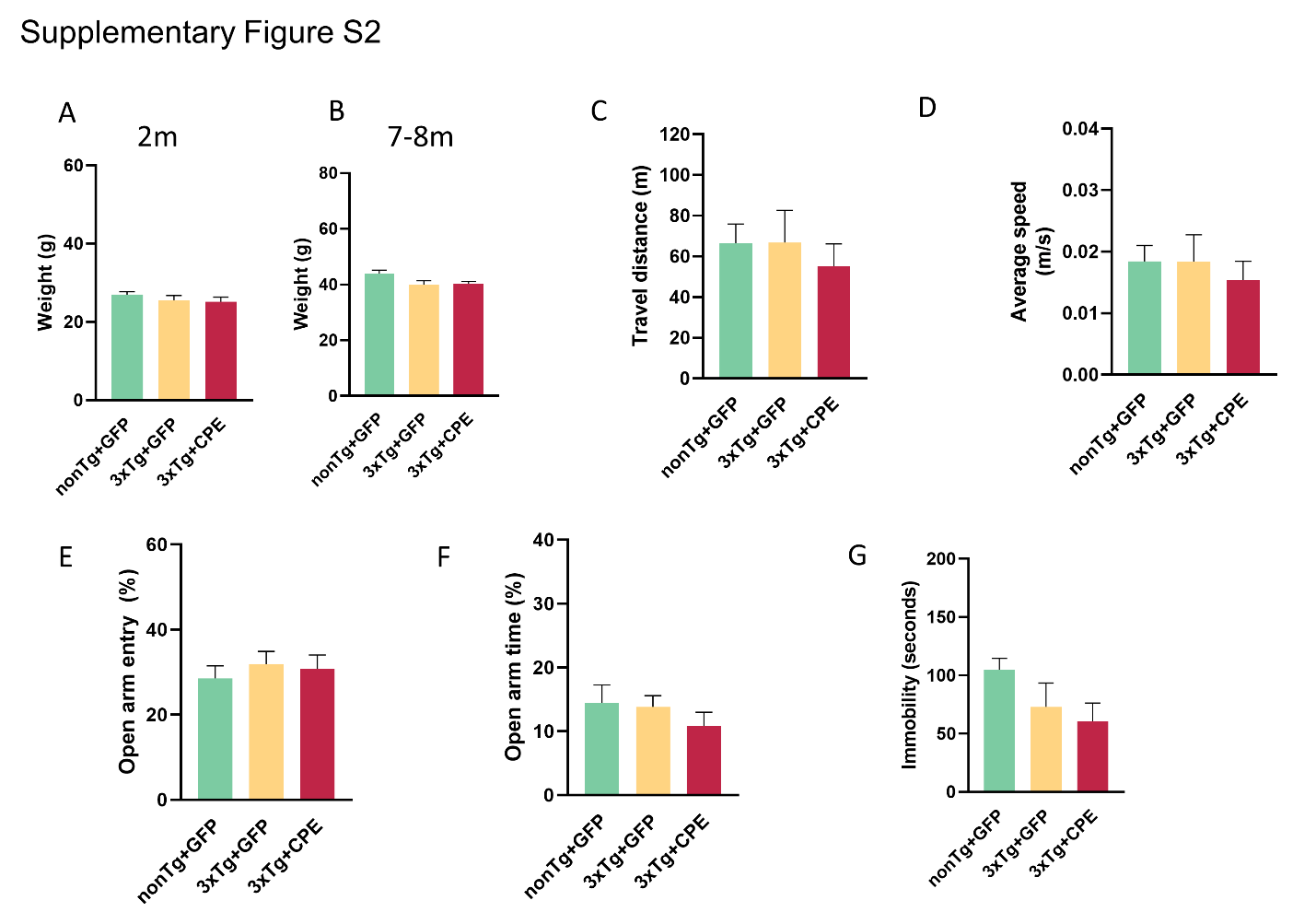
**

(A) Weight of mice at age of 2 months and (B) 7-8 months Hippocampal overexpression of CPE did not induce any significant changes in weight in 3xTg+GFP and 3xTg+CPE mice in comparison with nonTg+GFP mice. One-way ANOVA analysis followed by Tukey’s post-hoc multiple comparison test. n=12 for nonTg+GFP, n=11 for 3xTg+GFP, n=14 for 3xTg+CPE. Values are mean ± SEM.

(C) Travel distance of nonTg+GFP, 3xTg+GFP and 3xTg+CPE mice in open field test. One-way ANOVA analysis followed by Tukey’s post-hoc multiple comparison test, n=12 for 3xTg+GFP, n=13 for 3xTg+CPE. The values are the mean ± SEM.

(D) Average travel speed of nonTg+GFP, 3xTg+GFP and 3xTg+CPE mice in open field test. One-way ANOVA analysis followed by Tukey’s post-hoc multiple comparison test. n=12 for nonTg+GFP, n=12 for 3xTg+GFP, n=13 for 3xTg+CPE. The values are the mean ± SEM.

(E) Open arm entry and (F) open arm time percentage of nonTg+GFP, 3xTg+GFP and 3xTg+CPE mice in elevated plus maze. One-way ANOVA analysis followed by Tukey’s post-hoc multiple comparison test. n=12 for nonTg+GFP, n=12 for 3xTg+GFP, n=14 for 3xTg+CPE. The values are the mean ± SEM.

(G) Immobility time of nonTg+GFP, 3xTg+GFP and 3xTg+CPE mice in forced-swim test. One-way ANOVA analysis followed by Tukey’s post-hoc multiple comparison test. n=12 for nonTg+GFP, n=10 for 3xTg+GFP, n=13 for 3xTg+CPE. The values are the mean ± SEM.

**Figure S3**

**
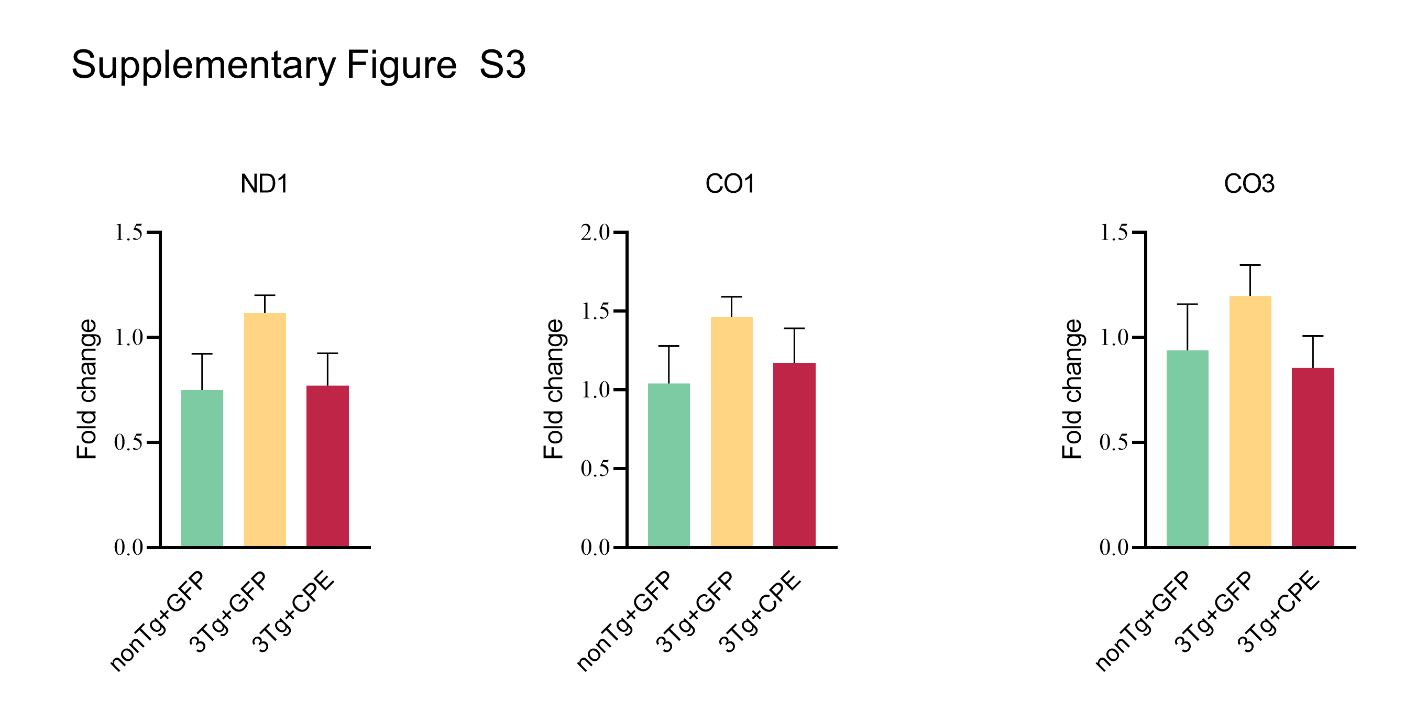
**

Analysis of mitochondrial metabolism gene expression in nonTg+GFP, 3xTg+GFP and 3xTg+CPE mouse hippocampus. Bar graphs showing fold change of *ND1, CO1* and *CO3* mRNAs in these mice . Values are mean ± SEM. n=5.

**Figure S4**

**
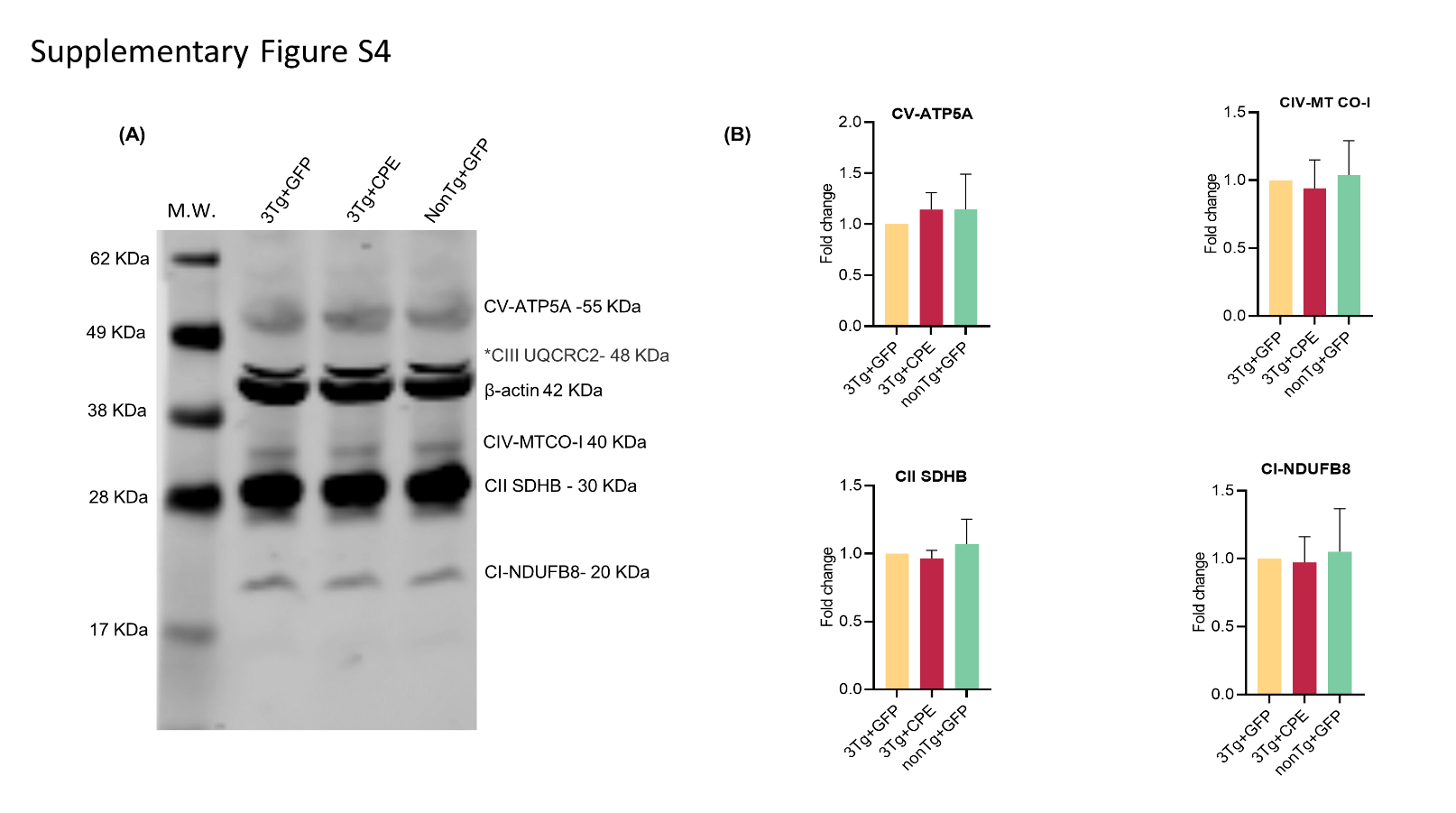
**

**(A)** Western blot **(B)** densitometric analysis of various proteins related to metabolism pathway. Total OXPHOS Rodent WB Antibody Cocktail (ab110413) was used 1:1000 overnight at 4°C. Bands were normalized to internal control β-actin. *CIII UQCRC2- 48 Kda could not be calculated from 3 animals so densitometric analysis was not included.

**Figure S5**


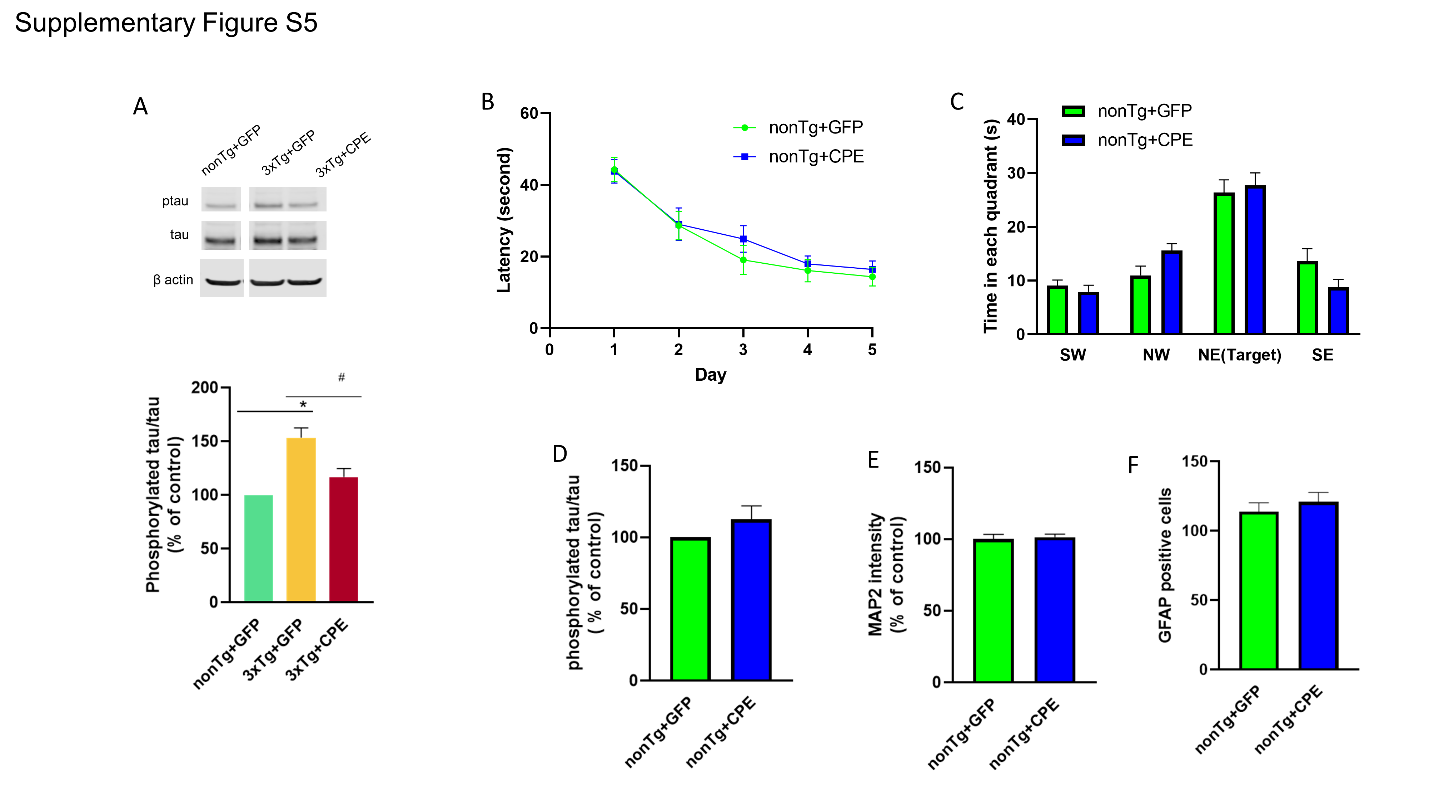


(A) Representative Western blot and quantification of phosphorylated Tau (Ser202, Thr205) expression in hippocampus of nonTg+GFP, 3xTg+GFP and 3xTg+CPE mice at age ~8months. Phosphorylated tau was increased in hippocampus of 3xTg+GFP in comparison with nonTg+GFP, * p=0.0004; overexpression of CPE in 3xTg+CPE mice significantly reduced phosphorylated tau, ~~#~~p=0.0082 One-way ANOVA analysis followed by Tukey’s post-hoc multiple comparison test, [F (_2,12_) =15.14, p=0.0005]. n=5 mice per genotype. Values are mean ± SEM.

(B) Overexpression of CPE in nonTg mice did not induce any changes in learning. n=12 for nonTg+GFP, n=15 for nonTg+CPE. Values are mean ± SEM.

(C) Over expression of CPE in nonTg mice did not induce any significant changes in time spent in the target area of Morris water maze. nonTg+GFP: n=12; nonTg+CPE: n=15. Values are mean ± SEM.

(D) Quantification of phosphorylated tau expression in the hippocampus of nonTg+GFP and nonTg+CPE mice at age of ~8 months. Phosphorylated tau was not changed in the hippocampus of nonTg+GFP in comparison with nonTg+CPE. t-test. n=5 mice per genotype. The values are the mean ± SEM.

(E) Quantification of MAP2 intensity in CA1 of nonTg+GFP and nonTg+CPE mice at age ~8month. No significant differences were found between two groups. t-test. n=6 per genotype. The values are the mean ± SEM.

(F) Quantification of GFAP positive cells in CA1 of nonTg+GFP and nonTg+CPE mice at age ~8months. No significant differences were found between two groups. t-test. n=6 per genotype. The values are the mean ± SEM.

**Supplementary Tables**

**Table S1. List of antibodies for Western Blot**

|  | | | | |
| --- | --- | --- | --- | --- |
| Antibody | Host | Dilution | Catalog number | Source |
| CPE | mouse | 1:2000 | 610759 | BD Biosciences |
| β-actin | Rabbit | 1:3000 | mAb #4970s | Cell signaling |
| β-actin | Mouse | 1:3000 | A5316 | Sigma Aldrich |
| GFP | Rabbit | 1:3000 | ab183734 | Abcam |
| GFAP | Rabbit | 1:2000 | Z0334 | Dako Agilent |
| CD11b/c | Rabbit | 1:1000 | bs-1014R | Bioss |
| ptau (PHF-13) | mouse | 1:1000 | sc-32275 | Santa Cruz |
| ptau(AT8) | mouse | 1:1000 | MN1020 | ThermoFisher |
| tau | Mouse | 1:2000 | sc-32274 | Santa Cruz |
| APP | Rabbit | 1:2000 | ab32136 | Abcam |
| hAPP | mouse | 1:500 | YA366018 | Invitrogen |
| Card14 | Mouse | 1:1000 | ab168096 | Abcam |
| Plin4 | Rabbit | 1:1000 | NBP2-13776 | Novusbio |
| Bcl2 | Mouse | 1:2000 | 15071 | Cell signaling |
| Bax | Rabbit | 1:2000 | #2772 | Cell signaling |
| proBDNF | mouse | 1:2000 | sc-65514 | Santa Cruz |
| Serpina3g | Rabbit | 1:1000 | TA363307 | OriGene |

**Table S2. List of Antibodies for immunohistochemistry staining**

|  | | | | |
| --- | --- | --- | --- | --- |
| Antibody | Host | Dilution | Catalog number | Source |
| CPE | Rabbit | 1:5000 | pa5-101497 | ThermoScientific |
| GFAP | Rabbit | 1:1500 | z0334 | Dako |
| DoubleCortin | Rabbit | 1:1500 | 4604s | Cell Signaling |
| APP | Rabbit | 1:3000 | 51-2700 | ThermoScientific |
| MAP 2 | Chicken | 1:1500 | cpca-map2 | EnCor |
| CD68 | Rabbit | 1:15000 | 600-401-R10 | Rockland |
| Anti-Rabbit Cy3 | Donkey | 1:500 | 711-165-152 | Jackson Lab |
| Anti-Chicken Cy3 | Donkey | 1:500 | 703-165-155 | Jackson Lab |
| Anti-Rabbit Biotinylated | Goat | 1:1000 | ba-1000 | Vector |

**Table S3. Transcription factor primers**

TaqMan Human Transcription Factors array was used to assess different transcription factors. HEK293 cells were transiently transfected with CPE or vector for 48 hours before harvest. Real time PCR was used to determine relative mRNA expression level.

| Gene | Fold change |
| --- | --- |
| AR | 0.573177 |
| ARNT | 1.052185 |
| ATF1 | 0.970304 |
| ATF2 | 1.703922 |
| ATF3 | 0.256801 |
| ATF4 | 1.377818 |
| CEBPA | 0.650008 |
| CEBPB | 0.654996 |
| CEBPG | 0.480117 |
| CREB1 | 1.213178 |
| CREBBP | 0.31918 |
| CTNNB1 | 1.144813 |
| DR1 | 1.187683 |
| E2F1 | 0.860088 |
| E2F6 | 1.861388 |
| EGR1 | 0.332453 |
| ELK1 | 1.164351 |
| ESR1 | N/A |
| ETS1 | 0.490702 |
| ETS2 | 0.536283 |
| FOS | 0.335091 |
| FOXA2 | 0.333855 |
| FOXO1 | 1.23678 |
| GATA1 | N/A |
| GATA2 | 0.737859 |
| GATA3 | 0.363003 |
| GTF2B | 1.449518 |
| GTF2F1 | 0.79069 |
| HAND1 | 0.192968 |
| HAND2 | 0.709276 |
| HDAC1 | 0.891394 |
| HIF1A | 4.080272 |
| HNF4A | 0.225187 |
| HOXA5 | 1.456711 |
| HSF1 | 0.494886 |
| ID1 | 2.202684 |
| IRF1 | 0.294944 |
| JUN | 1.056846 |
| JUNB | 2.232453 |
| JUND | N/A |
| MAX | 0.547862 |
| MEF2A | 0.517048 |
| MEF2B | 0.470462 |
| MEF2C | 0.760428 |
| MYB | 2.568465 |
| MYC | 0.782932 |
| MYF5 | N/A |
| MYOD1 | N/A |
| NFAT5 | 1.160234 |
| NFATC1 | 0.660481 |
| NFATC2 | 1.15321 |
| NFATC3 | 0.456909 |
| NFATC4 | 0.69309 |
| NFKB1 | 1.22623 |
| NFYB | 1.390609 |
| NR3C1 | 0.829802 |
| PAX6 | 1.013712 |
| POU2AF1 | N/A |
| PPARA | 1.050552 |
| PPARG | 1.503882 |
| RB1 | 1.870672 |
| REL | 0.64557 |
| RELA | 0.415561 |
| RELB | 0.669706 |
| SMAD1 | 0.61101 |
| SMAD4 | 1.198992 |
| SMAD5 | 0.449439 |
| SMAD9 | 0.84289 |
| SP1 | 0.55471 |
| SP3 | 0.846234 |
| STAT1 | 2.354891 |
| STAT2 | 0.646641 |
| STAT3 | 0.748597 |
| STAT4 | 1.287888 |
| STAT5A | 0.363513 |
| STAT5B | 0.943026 |
| STAT6 | 0.521109 |
| TBP | 1.416564 |
| HNF1A | N/A |
| TCF7L2 | 1.304614 |
| TFAP2A | 1.543406 |
| TGIF1 | 0.779058 |
| TP53 | 0.901527 |
| YY1 | 1.070727 |

**Table S4. qPCR validation primers for human HEK293 and mouse HT22 cells**

| Human GAPDH primer | | |  |  |
| --- | --- | --- | --- | --- |
| Forward: 5’-GTCTCCTCTGACTTCAACAGCG-3’ | | | | |
| Reverse: 5’-ACCACCCTGTTGCTGTAGCCAA-3’ | | | | |
|  |  |  |  |  |
| Mouse GAPDH primer | | |  |  |
| Forward: 5’-CATCACTGCCACCCAGAAGACTG-3’ | | | | |
| Reverse: 5’-ATGCCAGTGAGCTTCCCGTTCAG-3’ | | | | |
|  |  |  |  |  |
| Mouse SP1 primer | | |  |  |
| Forward: 5’- CTCCAGACCATTAACCTCAGTGC-3’ | | | | |
| Reverse: 5’- CACCACCAGATCCATGAAGACC-3’ | | | | |
|  |  |  |  |  |
| Human SP1 primer | | |  |  |
| Forward: 5’- AGGACTACGTGGAGATCAACGG-3’ | | | | |
| Reverse: 5’-TCACTGGAGTCGTAGGAGAGGT-3’ | | | | |
| Human CREBBP primer | | |  |  |
| Forward: 5’- AGTAACGGCACAGCCTCTCAGT-3’ | | | | |
| Reverse: 5’- CCTGTCGATACAGTGCTTCTAGG-3’ | | | | |
|  |  |  |  |  |
| Mouse CREBBP primer | | |  |  |
| Forward: 5’- CACCATCTGTGGCTACTCCTCA-3’ | | | | |
| Reverse: 5’- GGTTTCAGCACTGGTCACAGAG-3’ | | | | |
|  |  |  |  |  |
| Human HSF-1 primer | | |  |  |
| Forward: 5’- TGAAAAGTGCCTCAGCGTAGCC -3’ | | | | |
| Reverse: 5’- TGCTCAGCATGGTCTGCAGGTT-3’ | | | | |
|  |  |  |  |  |
| Mouse HSF-1 primer | | |  |  |
| Forward: 5’- GCACACTCTGTGCCCAAGTATG-3’ | | | | |
| Reverse: 5’- AGCTGGTGACAGCATCAGAGGA-3’ | | | | |
|  |  |  |  |  |
| Human SMAD5 primer | | |  |  |
| Forward: 5’- CAGGAGTTTGCTCAGCTTCTGG-3’ | | | | |
| Reverse: 5’- GGTGCTGGTTACATCCTGCCG-3’ | | | | |
|  |  |  |  |  |
| Mouse SMAD5 primer | | |  |  |
| Forward: 5’- CAGGAGTTTGCTCAGCTTCTGG -3’ | | | | |
| Reverse: 5’- ACGTCCTGTCGGTGGTACTCTG-3’ | | | | |
| Mouse Serpina3g primer | | |  |  |
| Forward: 5’-GCATCAGGGAAGTCTTCTCCAC-3’ | | | | |
| Reverse: 5’-CACAACATCCGACACCTGCCAT-3’ | | | | |
|  | | |  |  |
| Human APP primer | | |  |  |
| Forward: 5’-CCTTCTCGTTCCTGACAAGTGC-3’ | | | | |
| Reverse: 5’-GGCAGCAACATGCCGTAGTCAT-3’ | | | | |
|  |  |  |  |  |
| Mouse APP primer | | |  |  |
| Forward: 5’-TCCGTGTGATCTACGAGCGCAT-3’ | | | | |
| Reverse: 5’-GCCAAGACATCGTCGGAGTAGT-3’ | | | | |

**Table S5. TaqMan™ probes for mitochondrial metabolism genes**

| MT-ND1 | Hs02596873 |
| --- | --- |
| MT-ND4 | Hs02596876 |
| MT-ND6 | Hs02596879 |
| MT-CO1 | Hs02596864 |
| MT-CO3 | [Hs02596866](https://www.thermofisher.com/taqman-gene-expression/product/Hs02596866_g1?CID=&ICID=&subtype=) |
| MT-ATP6 | Hs02596862 |
| ACTB | Hs03023880 |
| HPRT1 | Hs02800695 |

**Reference**

1. Holmes, A., Kinney, J.W., Wrenn, C.C., Li, Q., Yang, R.J., Ma, L., Vishwanath, J., Saavedra, M.C., Innerfield, C.E., Jacoby, A.S., et al. (2003). Galanin GAL-R1 receptor null mutant mice display increased anxiety-like behavior specific to the elevated plus-maze. Neuropsychopharmacology *28*, 1031-1044. 10.1038/sj.npp.1300164.

2. Cheng, Y., Cawley, N.X., Yanik, T., Murthy, S.R., Liu, C., Kasikci, F., Abebe, D., and Loh, Y.P. (2016). A human carboxypeptidase E/NF-alpha1 gene mutation in an Alzheimer's disease patient leads to dementia and depression in mice. Transl Psychiatry *6*, e973. 10.1038/tp.2016.237.

3. Cheng, Y., Rodriguiz, R.M., Murthy, S.R., Senatorov, V., Thouennon, E., Cawley, N.X., Aryal, D.K., Ahn, S., Lecka-Czernik, B., Wetsel, W.C., and Loh, Y.P. (2015). Neurotrophic factor-alpha1 prevents stress-induced depression through enhancement of neurogenesis and is activated by rosiglitazone. Mol Psychiatry *20*, 744-754. 10.1038/mp.2014.136.
